# Supplementary material for: A fast comparative genome browser for diverse bacteria and archaea
Source: PLoS One. 2024 Apr 9;19(4):e0301871. doi: 10.1371/journal.pone.0301871 (PMC11003636; doi:10.1371/journal.pone.0301871)
Supplement: Attachment — Submitted filename: responses.pdf [file pone.0301871.s001.pdf]

Responses to comments from reviewer #1

Reviewer 1: "One computational limitation is rapid identification of homologs in such vast datasets of thousands of genera and species. Thus, the authors use MMseqs2, which is a fast alternative to Blast. How does MMSeqs2 compare to DIAMOND (Buchfink et al., 2015, PMID: 25402007)? From DIAMOND's web site "DIAMOND is a high-throughput program for aligning DNA reads or protein sequences against a protein reference database such as NR, at up to 20,000 times the speed of BLAST, with high sensitivity."

DIAMOND is optimized for searches with many queries. For searches with a single query, as would occur during interactive use, our experience is that DIAMOND takes around the same time as protein BLAST.

Reviewer 1: "The authors apply a homology cutoff of 1e-3. From my experience, I see better results with 1e-5. 1e-3 tends to get a lot of false positives, but this is only a comment of mine."

The editor was also curious about this question, and the related issue of how fast.genomics handles compositional bias. To address it, we added a section to the Materials and Methods, titled "E-values and compositional bias". Briefly, we do not believe that there are many false positives in the hits with e-values near the cutoff.

We'd also like to note that for most queries, changing the e-value cutoff from 1e-3 to 1e-5 would not affect the gene neighborhood view with default settings. For the main database, the gene neighborhood view includes the top 50 hits by default. In the test set of 1,000 random prokaryotic proteins, only 8% have any hits from MMseqs2 with rank <= 50 and E > 1e-5 (and E < 1e-3). For the test set of 1,000 proteins from Rhizobiales, only 6% have hits from clustered search with rank <= 100 and E > 1e-5. (The default view for order databases shows species clusters of genes, and there's ~2x more genomes than species in this order-level database, so it is more appropriate to consider the top 2*50 = 100 homologs.)

More broadly, the focus of fast.genomics is on homologs which are likely to have the same function, namely closer homologs or homologs that have the same gene context (and are unlikely to be false positives).

Reviewer 1: "Since different researchers have their own criteria for homology, depending on what they are after, it would be good to have a local version where the user may define their sets of genomes and their homology criteria, such as e-value and % identity with certain coverage."

Fast.genomics allows the user to download a table of homologs. We revised the Results to mention that this table includes e-values. Similarly, the gene presence/absence tool allows the user to download a table of the top hit for both queries in each genome; this table now includes the e-values. In either case, the user could easily filter out weak hits from these tables, or choose a subset of genomes of interest.

If a user wishes to build a version of fast.genomics with additional genomes, the source code for fast.genomics is available, including the scripts for building the database. We revised the code

availability statement to make it clear that the scripts for building the databases are included.

Reviewer 1: "Concerning which genomes to include: I understand that the authors use the species name as provided by genebank. However, very frequently, genomes are misannotated in terms of species names (Nikolaidis et al., 2022 and Nikolaidis et al., 2023 - PMID: 36144322 and PMID: 37266990). One approach, maybe for future updates of the web-tool is to use FASTANI…"

Actually, all taxonomic assignments in fast.genomics, including the species names, are taken from GTDB, which uses ANI comparisons to define species. We revised the section of the Results on "The fast.genomics databases" to explain that the species definitions in fast.genomics are from GTDB.

Responses to comments from reviewer #2

Reviewer 2: "1) I feel that the authors have not performed a complete and adequate comparison with other competing tools. Especially with some tools that provide an integrated environment for analysis, management, storage, and sharing of metagenomic projects, like IMG/M or MGnify (supported by the EBI). Although, they perform some form of assessment using a specific example, namely the BT2172 sequence, I would have liked to see a more thorough comparison with tools such as IMG/M. These tools are specifically built for running large jobs and are hosted in servers with high calibre specifications suited for fast running and high sensitivity of results."

In regards to "These tools are ... suited for fast running and high sensitivity of results", this is not our experience. For instance, the IMG/M web site states that "Real time BLAST request on average takes about 2 mins. to 15 mins. to complete." The homologs feature of IMG/M is just not designed to be fast. And, as discussed in our manuscript, we feel that the IMG web site does not organize the results as well as fast.genomics does. Similarly, our impression is that MGnify is not suitable for fast searches. As of February 13, the sequence search page says: "We recognize that our service has faced challenges in providing the latest version of the MGnify protein database, and we sincerely apologize for any inconvenience caused. The recent rapid growth of the protein database to over 3 billions has present technical challenges in scaling the search infrastructure which we are currently addressing."

We did not compare fast.genomics to MGnify because MGnify does not provide analogous functionality. In particular, as far as we know, MGnify does not provide any way to compare the gene neighborhoods of the homologous proteins or to compare the presence/absence of two proteins across taxa.

More broadly, the reviewer was concerned that we did not compare fast.genomics to tools that support metagenomics projects -- but supporting metagenomics might not be compatible with the goals of fast.genomics. In particular, fast.genomics includes only high-quality genomes (whether from isolates or assembled from metagenomes) to ensure that analyses of the presence or absence of a gene family, across genomes will give reliable results. It's not clear how to compare gene presence/absence across taxa from fragmented metagenomic assemblies. We do hope that in the future, many more high-quality MAGs will be available, and fast.genomics’ coverage of the diversity of bacteria and archaea will improve (see the Conclusions section).

In the revised manuscript, the section on "The fast.genomics databases" clarifies why we only include high-quality genomes. And a new paragraph in the "Limitations" section reports how many MAGs are included and discusses the trade-off between supporting presence/absence analyses and incorporating more of the sequenced diversity of bacteria and archaea.

Reviewer 2: "2) Recently Pavlopoulos et al. Nature, 2023 (Unraveling the functional dark matter through global metagenomics) published an approach that examines functional diversity beyond what was currently possible through the lens of reference genomes. Their computational approach generates reference-free protein families from the sequence space in metagenomes. They analysed over 26,000 metagenomes and identified >1 billion protein sequences with no prior similarity to any sequences from >100,000 reference genomes or the Pfam database. It would be nice for the authors to make a comparison with this approach and provide at least some measure of sensitivity with the results reported by this publication. Moreover, Pavlopoulos et al. provided a tremendous amount of novel protein families which have not been considered by the authors in their approach."

Fast.genomics does not use precomputed families, and we demonstrated that for the main database (of mostly isolate genomes), this often leads to better results. So we're not sure if it would make sense to incorporate the families identified by Pavlopoulos et al into fast.genomics.

Fast.genomics's main database does include 1,418 non-isolate genomes, so some of the families from Pavlopoulos et al do have homologs in fast.genomics already, and these can be found using fast.genomics's search tools. As metagenome assemblies improve, a greater proportion of the diversity of bacteria and archaea will be represented in fast.genomics. For now, fast.genomics's databases do not include low-quality MAGs because we want to support presence/absence analyses (see above).
